# Protective Effects of Aucubin in DSS-Induced Colitis: Modulation of Inflammatory Pathways, Intestinal Barrier Integrity, and Gut Microbiota

**DOI:** 10.3390/foods14213648

**Published:** 2025-10-26

**Authors:** Yong Zhang, Han Qiao, Yuxin Cao, Meng Zhang, Xuelei Zhang, Peng Li

**Affiliations:** 1 Institute for Biological Engineering, Henan University of Technology, Zhengzhou 450001, China; yongzhang208@haut.edu.cn (Y.Z.); qiaohan137@126.com (H.Q.); caoyuxin0611@163.com (Y.C.); 18567152172@163.com (M.Z.); zhangxuelei1020@126.com (X.Z.); 2 Institute for Complexity Science, Henan University of Technology, Zhengzhou 450001, China

**Keywords:** *Eucommia ulmoides*, aucubin, DSS-induced colitis, gut microbiota, inflammation

## Abstract

As an active ingredient in Eucommia leaf, aucubin (AU) is natural and safe, and studies have shown that aucubin (AU) demonstrates great potential in its anti-inflammatory, antioxidant, neuroprotective, and anti-osteoporotic properties. However, AU has been less studied in colitis. In this experiment, we used DSS-induced mice to establish a colitis model to investigate the ability of AU to alleviate colitis. The results show that, in animal experiments, AU increased body weight, reduced disease activity index (DAI) scores and organ indices, restored colon morphology, and increased superoxide dismutase (SOD), glutathione peroxidase (GSH-PX), and catalase (CAT) levels in mouse serum and colon. It also reduced malondialdehyde (MDA) levels, decreased the relative mRNA expression levels of inflammatory factors *IL-1β*, *TNF-α*, *IL-18*, *MyD88*, and *NF-κB*, and increased the relative mRNA expression levels of intestinal barrier-related genes *OCLN*, *CLDN1*, *CLDN2*, *ZO-2*, and *MUC1*. AU also upregulated the abundance of bacterial groups such as *Bacteroidota*, *Firmicutes*, and *Verrucomicrobiota*, and downregulated the abundance of bacterial groups such as *Proteobacteria* and *Deferribacterota*, thereby regulating the intestinal microbiota. In cell experiments, AU increased the relative mRNA expression levels of intestinal barrier-related genes *MUC2*, *ZO-1*, *OCLN*, and *CLDN1*, reduced the relative expression levels of inflammatory factors *IL-1β* and *TNF-α*, and increased the relative expression level of the anti-inflammatory factor *IL-10*. Additionally, AU significantly reduced the relative expression levels of *IL-1β*, *IL-1R*, *MyD88*, *TAK1*, *IKKα*, and *RelA*. This study provides a theoretical and technical basis for the large-scale preparation of aucubin and the alleviation of inflammatory bowel disease.

## 1. Introduction

Inflammatory bowel disease (IBD) constitutes a persistent inflammatory disorder marked by the disturbance of gut homeostasis, culminating in dysbiosis within the intestinal ecosystem [1]. Broadly speaking, IBD encompasses both specific forms with known etiology and non-specific forms with unknown etiology. Specific forms of IBD encompass infectious colitis, ischemic colitis, drug-related colitis, and radiation-associated colitis. Converse-ly, non-specific variants include ulcerative colitis (UC), Crohn’s disease (CD), and un-complicated ulcers [2].UC, in particular, is confined to the colonic mucosa and submucosa, manifesting as symptoms including bloody diarrhea, abdominal pain, and weight loss. Globally, UC affects an estimated 5–10 million individuals, with incidence rates rising annually by 5–10% in both developed and emerging economies, driven by factors such as Western-ized diets, urbanization, and environmental stressors. This escalating burden imposes substantial healthcare costs and diminishes quality of life, underscoring the urgency for novel therapeutic strategies [3]. Currently, conventional treatments for colitis typically involve the use of medications such as aminosalicylates, corticosteroids, and antibiotics [4]. Beyond standard treatments, alternative approaches—such as leukocytapheresis, in-organic nitrites or nitrates, and fecal microbiota transplantation (FMT)—have been investigated for managing UC [5,6,7]. However, these drugs can cause a range of side effects in clinical settings, such as im-munosuppression leading to opportunistic infections, and metabolic disturbances (e.g., osteoporosis from steroids). These limitations highlight the need for safer, mecha-nism-based interventions derived from natural products, which offer multi-target ef-ficacy with potentially fewer adverse effects. Preclinical models are pivotal in bridging this gap, with the dextran sulfate sodium (DSS)-induced colitis model in mice being a cornerstone for UC research. This acute model recapitulates key histopathological fea-tures of human UC, Its reproducibility and ethical feasibility have facilitated the eval-uation of numerous candidate com-pounds, paving the way for translational studies. Besides, Owing to the anti-inflammatory and antioxidative attributes of bioactive compounds from plants, such natural agents operate via diverse pathways, such as inhibiting TNF-α, IL-1β, MDA, and nuclear factor κB (NF-κB). As a result, more and more people are turning to natural plant extracts as the preferred treatment for UC [8,9,10].

Eucommia ulmoides Oliv. (Eucommiaceae) is a deciduous tree, Native to central and southwestern China. As a source of aucubin—an iridoid glycoside with anti-inflammatory and antioxidant properties—the plant is predominantly harvested from cultivated plantations, where sustainable farming practices ensure consistent yields of bioactive compounds from leaves, bark, and seeds. *Eucommia ulmoides* is not only a traditional Chinese herbal medicine but also a leading example of dual-purpose plants that are both medicinal and edible. In recent years, it has been developed into functional food products [11]. Eucommia contains a variety of active compounds, including aucubin, chlorogenic acid, and flavonoids [12]. Aucubin, an iridoid glycoside (specifically a cyclopentanoid monoterpene derivative) found in the leaves of *Eucommia*, is a prominent compound in this plant and belongs to the iridoid glycoside class [13]. Aucubin features a distinctive cyclopentanopyran skeleton with a molecular formula of C_13_H_18_O_9_ and a molecular weight of 354.36 g/mol. Its chemical stability and bioavailability, enhanced by glycosidic linkages, contribute to its therapeutic potential. This compound (aucubin) is considered safe and non-toxic, and it exhibits various bioactive properties, such as antioxidative effects [14], neuroprotective [15], and anti-osteoporotic [16] effects, making it highly valuable for both food and pharmaceutical research and development. These attributes align with UC pathogenesis, where oxidative damage and NF-κB-mediated inflammation exacerbate mucosal injury.

This study established a DSS-induced IBD mouse model to evaluate aucubin’s impacts of aucubin on body weight, disease activity index (DAI), intestinal morphology, oxidative stress, gut microbiota, intestinal barrier function and inflammatory markers. In vitro, porcine small intestinal epithelial cells (IPEC-J2) were treated with DSS to induce inflammation, and the effects of aucubin on inflammatory markers, and intestinal barrier integrity were assessed. This study aims to provide a theoretical basis for the alleviation of inflammatory bowel disease by aucubin and to offer research insights for the development of functional foods.

## 2. Materials and Methods

### 2.1. Materials and Animals

Eucommia ulmoides foliage was procured from Henan Golden Eucommia Agricultural Science and Technology Co.Xuchang, China. Eucommia glycosides were extracted using an ethanol concentration of 24%, a solid-to-liquid ratio of 1:28 (g/mL), a temperature of 71 °C, and a duration of 34 min. Dextran sulfate sodium (DSS) was obtained from Shanghai McLean Biochemical Technology Co., Ltd., Shanghai, China; AU standard samples were sourced from Shanghai Yuanye Biotechnology Co., Ltd., Shanghai, China; and the porcine small intestinal epithelial cell line (IPEC-J2) was purchased from Shanghai Hongshun Biotechnology Co., Ltd., Shanghai, China. DMEM medium and fetal bovine serum (FBS) were both acquired from Beijing Solarbio Technology Co., Ltd., Beijing, China; PBS (phosphate-buffered saline) was purchased from Wuhan Savier Biotechnology Co., Ltd., Wuhan, China; and the CCK-8 assay kit was obtained from Shanghai Biyuntian Biotechnology Co., Ltd., Shanghai, China. Thirty 4-week-old male Kunming mice were used for the experiments and were purchased from Henan Skebes Biotechnology Co, Zhengzhou, China. All animal procedures complied with the ethical guidelines and protocols endorsed by the Institutional Animal Care and Use Committee of Henan University of Technology (HAUTETHI2022-0723).

### 2.2. Animal Experiment Design

Thirty 4-week-old male Kunming mice were used in this study, purchased from Henan Scibes Biological Science and Technology Co., Ltd., Alameda, CA, USA, (Production License No.: SCXK (Yu) 2020-0005).

During the study, the animals were housed in conventional cages with a 12-h light-dark cycle, maintained at a temperature of 25 ± 0.5 °C and a humidity of 55 ± 2%, with unrestricted access to diet and drinking water. After a 1-week acclimatization period, the mice were randomly assigned to different groups: the normal group (CON), the model group (DSS), the low-dose AU group (LAU), the medium-dose AU group (MAU), and the high-dose AU group (HAU).

The experiment lasted a total of 28 days. From Day 1 to Day 7, all groups drank distilled water. The LAU, MAU, and HAU groups were treated with AU at doses of 40 mg/kg/d, 60 mg/kg/d, and 80 mg/kg/d, correspondingly, whereas the CON and DSS groups were administered an equivalent volume of sterile water. From Day 8 to Day 28, the DSS, LAU, MAU, and HAU groups were administered 3% DSS (dissolved in distilled water) intermittently to induce colitis. Mice received four cycles of DSS treatment, with each cycle consisting of 3 days of drinking 3% DSS, followed by 2 days of drinking distilled water. Throughout the modeling period, the LAU, MAU, and HAU groups continued to receive AU at the same doses, while the CON and DSS groups were given sterile water. Mice were euthanized on Day 29, and serum, liver, spleen, kidney, and colon tissues were collected for further analysis.

### 2.3. Assessment of Disease Activity Index (DAI)

During the modeling period, animal body weights were monitored daily, and fecal characteristics—such as occult or gross blood—were assessed per the protocol detailed by He BaoKun [17], with slight modifications. The Disease Activity Index (DAI) score was calculated as the sum of the percentage of weight loss, stool consistency, and stool bleeding scores. The DAI score was evaluated according to Table 1:

### 2.4. Determination of Length and Weight of Mouse Colon Tissue

A section of the colon, extending from the cecum to the anus, was collected. The 2 cm segment closest to the anus was excised, rinsed with saline, and the length of the colon was measured using a soft ruler. The colon segment was then weighed using an analytical balance.

### 2.5. HE Staining of Colon

A 0.5 cm segment of the middle colon tissue was placed in tissue fixative for fixation, followed by gradient ethanol dehydration, paraffin embedding, sectioning, and hematoxylin and eosin (HE) staining. The processed tissue was then examined under a microscope to observe morphological changes, and images were captured.

### 2.6. Determination of Organ Index

First, the mice were weighed. Next, the liver, spleen, and kidneys were carefully removed, and excess fat was trimmed away. The organs were then washed with saline, and any excess saline was absorbed using filter paper. Finally, the harvested organs were weighed. The organ index was calculated using the following formula:

Organ index = (Organ weight in mg)/(Body weight in g) × 100%

### 2.7. Determination of Oxidative Stress-Related Indexes

Mouse serum was collected by puncturing the eyeball, subsequently incubated at ambient temperature for 4 h. The serum was subsequently centrifuged at 4000 rpm for 15 min, and the supernatant was transferred to a −80 °C freezer for storage.

Levels of T-SOD, GSH-Px, MDA, CAT, and total protein in serum and colonic tis-sues were quantified per the protocols accompanying commercial assay kits (Nanjing Jiancheng Bioengineering Institute, Nanjing, China).

### 2.8. Real-Time Quantitative PCR Analysis (RT-q PCR)

Except for the colon tissue used for staining, the remaining colon samples were quickly frozen in liquid nitrogen and stored at −80 °C. A 0.5 mg portion of colon tissue was then removed from the −80 °C freezer, and the procedure was carried out according to the protocol provided by Nanjing Novozymes Biotechnology Co., Tianjin, China. RNA was extracted using an RNA extraction kit, and its concentration and quality were assessed using a NanoDrop 2000 spectrophotometer, Thermo Fisher Scientific, Waltham, MA, USA. Complementary DNA (cDNA) synthesis was conducted with a reverse transcription reagent kit. Real-time quantitative PCR (RT-qPCR) assays were carried out employing SYBR Green dye, utilizing β-actin as the reference gene for normalization. The mRNA levels of inflammatory factors and intestinal barrier-related genes were quantified using the 2^−ΔΔCt^ method. Primer sequences are provided in Table S1.

### 2.9. Determination of Microorganisms in the Cecum

A portion of the cecum from each mouse was collected and sent to Beijing Prime Biotech Co. (Beijing, China) for microbiological analysis.

### 2.10. Cell Culture and Treatment

#### Cell Resuscitation and Transfection

The cryopreserved IPEC-J2 cells were recovered from cryopreservation in liquid nitrogen and immediately thawed by immersion in a 37 °C water bath. Once fully thawed, the cell suspension was centrifuged, and the supernatant was carefully discarded. The cell pellet was then resuspended in fresh DMEM medium. After a second centrifugation under the same conditions, the cells were resuspended in complete growth medium containing DMEM, 10% fetal bovine serum (FBS), 100 U/mL penicillin, and 100 U/mL streptomycin. The cell suspension was plated in 25 cm^2^ cell culture flasks and maintained at 37 °C under a humidified atmosphere containing 5% CO_2_. The culture medium was replaced every 24 h. Once the cells reached 80–90% confluency, they were harvested by trypsinization for subculture.

### 2.11. Cell Experimental Grouping

To investigate the effects of AU standard and AU extract on DSS-induced cell inflammation, the cells were categorized into four groups:control (CON), DSS-induced (DSS) and AU extract-treated (AU extract). After pretreatment with 8 μg/mL of either AU standard or AU extract for 24 h, the cells were exposed to 2% DSS and incubated for 48 h to establish an inflammatory model for subsequent analyses.

### 2.12. RT-q PCR Analysis

After completing cell culture according to the test grouping described in Section 2.8, discard the culture medium and wash the cells twice with PBS. Once the PBS is removed, add 1 mL of FreeZol to lyse the cells in each well. Transfer the lysate into 1.5 mL centrifuge tubes. The subsequent procedures were carried out as described in section RT-qPCR. RNA was extracted from each experimental group, reverse-transcribed into cDNA, and analyzed by RT-qPCR. Data normalization was performed using GAPDH as the reference gene. The mRNA expression levels of intestinal barrier-related genes and inflammatory factors were calculated using the 2^−ΔΔCt^ method. Primer sequences are provided in Table S2.

### 2.13. Statistical Analysis

Data are presented as means ± standard error of the mean (SEM). All data were obtained from three or more independent experimental replicates and analyzed using IBM SPSS Statistics 26.0. Statistical significance was determined using one-way ANOVA followed by Duncan’s multiple range test. Statistical significance was determined at *p* < 0.05, with greater significance indicated at *p* < 0.01.

## 3. Results

### 3.1. Effect of AU on the Phenotype of Colitis Mice

**Figure 1:**
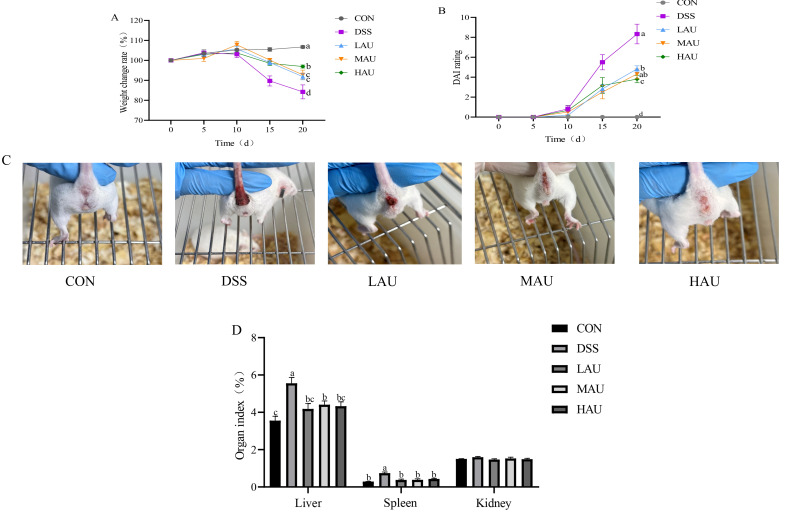
Apparent changes in mice in each group during modeling. (**A**) Weight change rate in body of mice. (**B**) DAI scores of mice. (**C**) Anal view of mice. (**D**) Organ indices of the liver, spleen, and kidneys of mice.The data are presented as mean ± SEM (*n* = 6). Different letters imply statistically significant differences at a level of *p* < 0.05.

To investigate the effect of AU on body weight in mice with DSS-induced colitis, body weight was monitored throughout the modeling period (Figure 1A). The CON group exhibited consistent weight gain over time. However, after the 10th day of mod-eling, all groups except the CON group displayed a downward trend in body weight. The most pronounced reduction occurred in the DSS group, with body weight plum-meting from 103.09% to 84.23%. However, mice in the LAU, MAU and HAU groups, to which AU was added, showed relief in the extent of weight reduction.

DAI is one of the main indexes to observe the success of the mouse model of DSS-induced colitis. The higher the DAI score, the more severe the degree of colitis in the mice. For this reason, during the modeling period, the body weight changes, the degree of diarrhea, and the degree of fecal bleeding of the mice in each group were observed and recorded, and scored with reference to the DAI scoring criteria (Table 1).

As shown in Figure 1B,C, during the modeling period starting from day 10, all groups except the CON group exhibited weight loss. The LAU, MAU, and HAU groups maintained normal fecal consistency with minor body weight fluctuations. On day 15, DSS group mice developed severe soft stools containing small amounts of blood, whereas the LAU, MAU, and HAU groups showed only mild stool softening. By day 20, DSS group mice displayed lethargy, severe diarrhea, perianal fecal adhesion, and grossly bloody stools. LAU group mice produced loose, moist stools with trace blood, while MAU group mice had severely soft stools containing slight blood. In contrast, HAU group mice passed loose but formed stools without blood. The body weight change rate and DAI scores demonstrated AU’s efficacy in alleviating colitis.

### 3.2. Effect of AU on Organ Indices in Colitis Mice

As depicted in Figure 1D, the DSS group resulted in a significant increase in the liver and spleen indices of colitis mice (*p* < 0.05). However, treatment with different doses of AU significantly reduced the liver and spleen indices (*p* < 0.05). In contrast, DSS-induced colitis had minimal effect on the kidney index. These findings demonstrate that AU mitigates the increase in liver and spleen indices, while having no effect on the kidney index.

### 3.3. Effect of AU on the Colon of Colitis Mice

**Figure 2:**
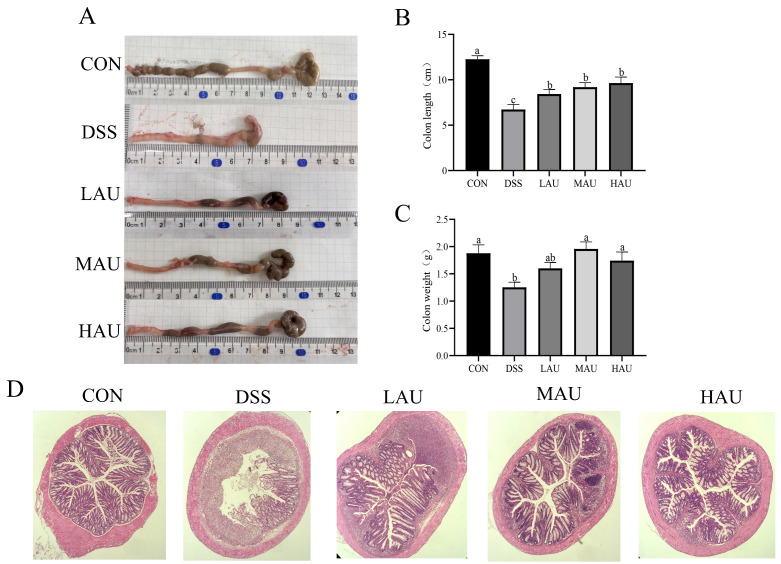
Changes in the colon of mice in each group. (**A**) Representative pictures of colon length in each group of mice. (**B**) Length of mouse colon in each group. (**C**) Weight of mouse colon in each group. (**D**) Representative results of H&E staining of mouse colon in each group (10×). The data are presented as mean ± SEM (*n* = 6). Different letters imply statistically significant differences at a level of *p* < 0.05.

In Figure 2A–C, mice in the CON group exhibited normal feces in the colon, with no signs of fecal impaction and typical coloration. In contrast, mice in the DSS group displayed significantly shortened colon length, abnormal fecal morphology, severe fecal impaction, and bright red colon coloration, indicative of hemorrhage. Compared to the CON group (colon length: 12.27 cm; colon weight: 1.88 g), the DSS group showed significantly shorter colon length (6.73 cm, *p* < 0.05) and lower colon weight (1.25 g, *p* < 0.05). Although the LAU group (colon length: 8.43 cm; colon weight: 1.60 g) did not show statistically significant differences compared to the DSS group, it exhibited a trend toward increased colon length and weight. Notably, both the MAU group (colon length: 9.20 cm; colon weight: 1.96 g) and HAU group (colon length: 9.64 cm; colon weight: 1.74 g) showed notable enhancements in colonic length relative to the DSS group, indicating that AU intervention substantially mitigated DSS-triggered colonic shortening and body mass loss in the murine model.

The degree of damage to the colon was assessed by HE staining.In Figure 2D, the CON group, the intestinal structure of the mice stayed preserved, with normal goblet cell populations and no evidence of inflammatory cell infiltration. In contrast, the DSS group displayed severe structural damage, characterized by the complete loss of goblet cells, ulcerations in the mucosal layer, and extensive inflammatory cell infiltration. These histopathological changes confirm the successful establishment of the experimental colitis model.

In contrast to the DSS group, the colonic tissues of the LAU group showed partial ulceration, with some recovery of goblet cells. The MAU group exhibited a reduction in colonic ulceration and mild inflammatory cell infiltration, with noticeable recovery of goblet cells. The HAU group demonstrated near-complete restoration of the normal colonic epithelial structure, significantly less ulceration and inflammatory cell infiltration, and nearly complete recovery of goblet cells.

### 3.4. Effect of AU on Oxidative Stress in Colitis Mice

InFigure 3A–D, the DSS group displayed notable decreases in serum GSH-Px, SOD, and CAT activities (*p* < 0.05), while MDA concentrations were significantly increased (*p* < 0.05) compared to the CON group, indicating severe oxidative stress in DSS-treated mice. Erum evaluations indicated no notable variations across the LAU, MAU, and HAU groups (*p* > 0.05), except for GPx activity. In colon tissue, however, AU treatment enhanced antioxidant enzyme activities and overall antioxidant capacity in all three treatment groups. Notably, AU intervention significantly increased SOD activity. Specifically, the HAU group exhibited a 62.9% increase in SOD activity (74.48 U/mL vs. 45.73 U/mL in the DSS group; *p* < 0.01).

**Figure 3:**
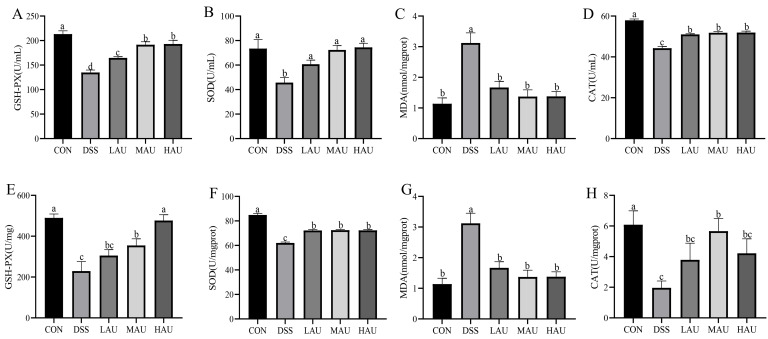
Changes in oxidative stress levels in mice in each group. Serum levels of (**A**) GSH-PX, (**B**) SOD, (**C**) MDA, and (**D**) CAT in each group of mice. Levels of (**E**) GSH-PX, (**F**) SOD, (**G**) MDA, (**H**) CAT in colon tissue of mice in each group. The data are presented as mean ± SEM (*n* = 6). Different letters imply statistically significant differences at a level of *p* < 0.05.

As shown in Figure 3E–H, the activities of GSH-PX and CAT in the colons of DSS group mice were significantly reduced (*p* < 0.05), while the levels of antioxidant enzymes and the antioxidant defense system were elevated to varying extents in the LAU, MAU, and HAU groups. Specifically, GSH-PX activity in the HAU group (476.62 U/mg) was significantly higher than in the DSS group (229.46 U/mg) (*p* < 0.01). Compared to the DSS group, MDA levels were significantly lower in the AU-treated groups (*p* < 0.05). These observations indicate that AU confers protective benefits in colitis-afflicted mice via boosting antioxidant enzyme functions, curbing lipid peroxidation, and alleviating oxidative stress.

### 3.5. Effect of AU on Inflammatory Factors in Colitis Mice

**Figure 4:**
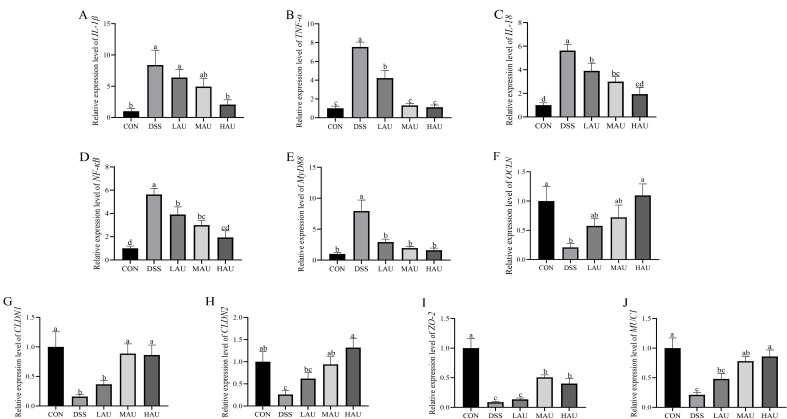
Expression of inflammatory factors in the colon of mice in each group. (**A**) IL-1β; (**B**) IL-18; (**C**) TNF-α; (**D**) NF-κB; (**E**) MyD88. Expression of colonic intestinal barrier-related genes in mice in each group. (**F**) OCLN; (**G**) CLDN1; (**H**) CLDN2; (**I**) ZO-2; (**J**) MUC1. The data are presented as mean ± SEM (*n* = 6). Different letters imply statistically significant differences at a level of *p* < 0.05.

As shown in Figure 4A–E, the relative mRNA expression of pro-inflammatory factors (*IL-1β*, *TNF-α*, and *IL-18*) was significantly increased in the DSS group compared to the CON group (*p* < 0.05), indicating a disruption in the balance of inflammatory factors and impaired intestinal immunity in the colitis mice. Compared to the DSS group, the HAU group exhibited significantly down-regulated expression of IL-1β (8.39 vs. DSS) and TNF-α (7.54 vs. DSS), with decreases of 75.4% and 85.1%, respectively (*p* < 0.05). Additionally, the mRNA expression of IL-1β and TNF-α was significantly lower in the MAU and HAU groups compared to the LAU group (*p* < 0.05).

Building on this, we examined the expression of inflammatory factor signaling pathways, including MyD88 and NF-κB, in the intestinal tissues of mice from each group. Compared to the CON group, the expression of MyD88 and NF-κB was significantly elevated in the DSS group (*p* < 0.01). However, in the HAU group, the expression of MyD88 and NF-κB was significantly reduced compared to the DSS group (*p* < 0.05). These results suggest that AU intake can down-regulate the expression of inflammatory factors in the colon, thereby protecting the colon from inflammatory damage.

### 3.6. Effect of AU on the Intestinal Barrier of Colitis Mice

In Figure 4F–J, the expression of *OCLN*, *CLDN1*, *CLDN2*, *ZO-2*, and *MUC1* was measured in this experiment. Quantitative detection by qRT-PCR revealed that the expression of *OCLN*, *CLDN1*, *CLDN2*, *ZO-2*, and *MUC1* was significantly reduced in the colon of mice in the DSS model group compared to the normal group. Specifically, the expression of *OCLN* and *CLDN2* was significantly decreased (*p* < 0.05), while the expression of *CLDN1*, *ZO-2*, and *MUC1* was significantly reduced (*p* < 0.01). Compared to the DSS group, the HAU group showed a significant increase in *CLDN1* and *ZO-2* (*p* < 0.05) and a highly significant increase in *OCLN*, *CLDN2*, and *MUC1* (*p* < 0.01).

### 3.7. Effect of AU on the Cecum Flora of Mice with Colitis

(1) Alpha Diversity Analysis

High-throughput sequencing generated 71,399 sequences in the CON group, 71,414 in the DSS group, 71,353 in the LAU group, 69,537 in the MAU group, and 70,527 in the HAU group. These accounted for approximately 89.18%, 89.20%, 89.18%, 89.38%, and 89.33% of the original sequences, respectively. The analysis revealed no significant differences in the α-diversity indices (ACE, Chao 1, Shannon, and Simpson) among the groups (*p* > 0.05).

(2) Beta Diversity Analysis

**Figure 5:**
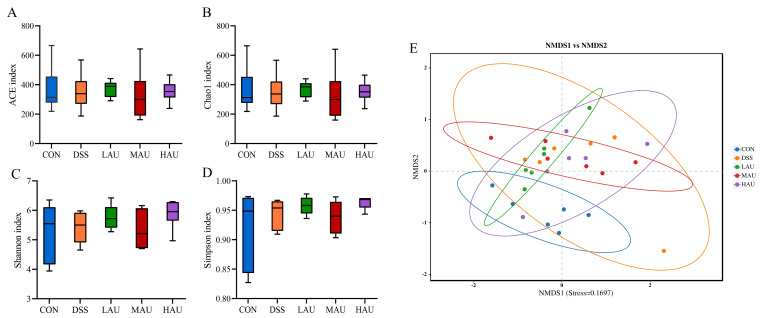
Analysis of microbial Alpha diversity in each group of mice. (**A**) ACE index; (**B**) Chao 1 index. (**C**) Shannon index. (**D**) Simpson index. (**E**) Analysis of microbial NMDS in each group of mice.

Beta diversity was used to assess the degree of similarity between the analyzed samples. NMDS was employed to evaluate the beta diversity, where the distance between points reflects the degree of dissimilarity; shorter distances indicate greater similarity in the composition of the two samples. As shown in Figure 5E, a clear separation was observed between the DSS group and the CON group, indicating that DSS induction significantly altered the intestinal microbiota of the mice compared to the CON group. However, the intake of AU modified the intestinal microbiota, with the HAU group partially overlapping with the CON group and showing a convergence toward the CON group. This suggests that AU supplementation improved the microbial diversity in mice with colitis.

(3) Analysis of Intestinal Flora Composition

**Figure 6:**
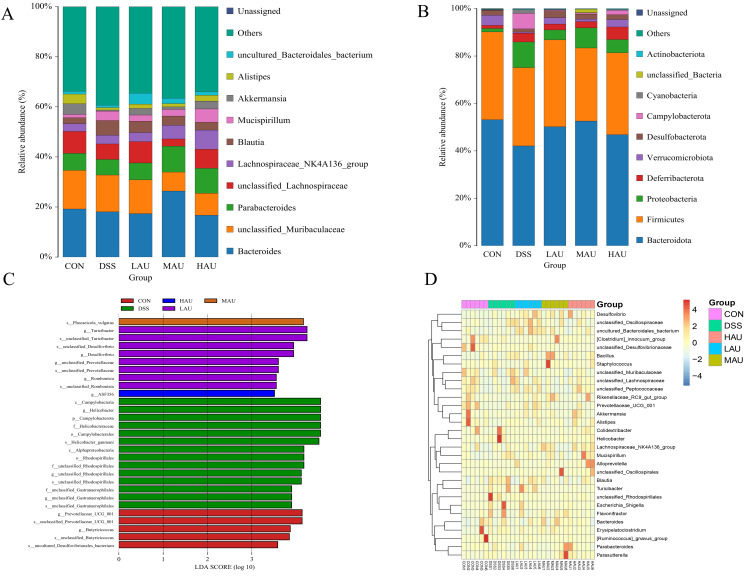
Analysis of microbial species composition of mice in each group. (**A**) Phyla with Top12 relative abundance of bacteria at the phylum level; (**B**) genera with Top12 relative abundance of bacteria at the genus level; (**C**) histogram of LDA distribution of gut microbiota; (**D**) genus-level clustering heat map.

The distribution and abundance of intestinal microbiota were analyzed in each group of mice. In Figure 6A, the top five phyla identified were *Bacteroidota*, *Firmicutes*, *Proteobacteria*, *Deferribacterota*, and *Verrucomicrobiota*. Compared with the CON group, the abundance of *Bacteroidota*, *Firmicutes*, and *Verrucomicrobiota* decreased in the DSS group, while the abundance of *Proteobacteria* and *Deferribacterota* increased. In the present study, the relative abundances of Bacteroidota in the LAU, MAU, and HAU groups were 50.19%, 52.58%, and 46.84%, respectively—all higher than the 42.09% observed in the DSS group. Conversely, the abundance of *Proteobacteria* in these groups (4.19%, 8.49%, and 5.60%) was lower than that in the DSS group (10.85%). Remarkably, the *Firmicutes/Bacteroidota* (F/B) ratio displayed an upward trajectory in the DSS group, while it declined in the MAU group.

In Figure 6B, at the genus level, the five dominant genera by relative abundance included *Bacteroides*, unclassified *Muribaculaceae*, *Parabacteroides*, unclassified_*Lachno-spiraceae*, and *Lachnospiraceae*_NK4A136 group. Relative to the CON group, the propor-tion of unclassified *Lachnospiraceae* declined in the DSS group, whereas the level of *Lachnospiraceae* NK4A136 group rose. Notably, the HAU group reversed this trend, showing an increase in unclassified_*Lachnospiraceae* and a decrease in *Lachnospiraceae*_NK4A136_group compared to the DSS group. These results suggest that AU may help ameliorate colitis in mice by modulating the gut microbiota composition.

(4) Intergroup Microbial Difference Analysis

Linear discriminant analysis (LDA) was used to estimate species with significant abundance differences between groups (LDA score > 3.5) in the gut microbiota of mice from each group. As shown in the LEfSe analysis in Figure 6C, the Prevotellaceae UCG 001, Butyricicoccus, and uncultured Desulfovibrionales bacteria were enriched in the CON group, while Campylobacter, Helicobacter, Rhodospirillales, and Gastranaerophilales were enriched in the DSS group. Turicibacter, Desulfovibrio, Prevotellaceae, and Rom-boutsia exhibited higher abundance in the LAU group, Phocaeicola_vulg_atus exhibited higher abundance in the MAU group, and Clostridium (ASF356) was enriched in the HAU group. Notably, Helicobacter pylori was not detected in the CON, LAU, MAU, or HAU groups. These results suggest that the LAU, MAU, and HAU groups were able to reduce the enrichment of Helicobacter pylori and mitigate the intestinal damage caused by DSS.

(5) Genus-Level Clustering Heatmap

Figure 6D displays the heatmaps depicting genus-level clustering patterns among the mouse cohorts. Figure 6DThe results indicate that the genera upregulated in the CON group include Desulfovibrionaceae, Alistipes, Akkermansia, Erysipelatoclostridium, and Ruminococcus. In the DSS group, the upregulated bacterial genera included *Helicobacter*, *Colidextribacter*, *Rhodospirillales*, and *Escherichia/Shigella*. After ingesting Eucommia glycoside, the upregulated bacterial genera included *Staphylococcus*, *Oscillospirales*, *Parasutterella*, *Clostridium_innocuum*_group, and *Mucispirillum*. *Helicobacter* pylori and *Escherichia/Shigella* are both gut microbiota associated with colorectal cancer, and the upregulation of these two genera can exacerbate IBD. Compared to the CON group, the DSS group showed a significant increase in harmful bacteria, and this effect was also observed in the group of mice that consumed Eucommia glycosides after DSS induction, leading to an increase in harmful bacteria such as Staphylococcus aureus. However, consuming Eucommia glycosides also influenced the intestinal microbiota by upregulating Oscillospirales. The Ruminococcus genus is known for producing short-chain fatty acids, such as butyrate, and is considered one of the key indicators of a healthy intestinal microbiota. This study found that DSS induction led to an increase in harmful bacteria in IBD mice, and after consuming Eucommia glycosides, the intestinal microbiota was modulated by upregulating the beneficial Ruminococcus genus.

### 3.8. Effect of AU on DSS-Induced Gut Barrier in IPEC-J2 Cells

**Figure 7:**
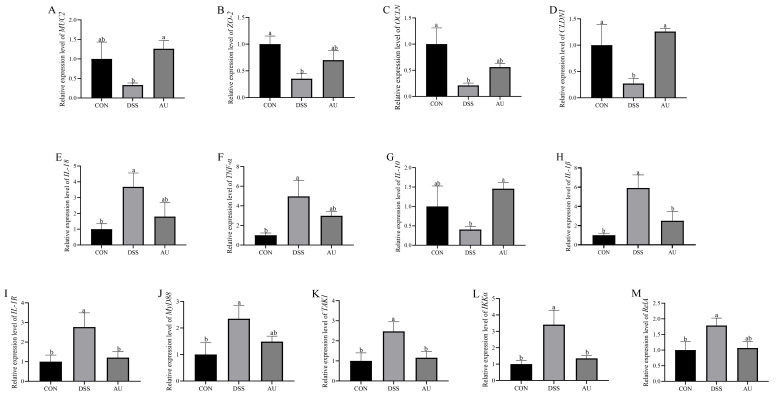
Effect of AU on the relative mRNA expression of gut barrier-related genes in IPEC-J2 cells. (**A**) MUC2. (**B**) ZO-1. (**C**) OCLN. (**D**) CLDN1. Effect of AU on relative mRNA expression of inflammatory factor-related genes in IPEC-J2 cells. (**E**) IL-18. (**F**) TNF-α. (**G**) IL-10. Effect of AU on the relative expression of genes related to MyD88/NF-κB signaling pathway in IPEC-J2 cells. (**H**) IL-1β (**I**) IL-1R. (**J**) MyD88. (**K**) TAK1. (**L**) IKKα. (**M**) RelA. The data are presented as mean ± SEM (*n* = 6). Different letters imply statistically significant differences at a level of *p* < 0.05.

In Figure 7A–D, the expression of *MUC2*, *ZO-1*, *OCLN*, and *CLDN1* was significantly reduced in IPEC-J2 cells compared to the CON group (*p* < 0.05). Compared with the DSS group, both the Standard AU and AU groups exhibited significantly increased expression of *MUC2*, *ZO-1*, *OCLN*, and *CLDN1* (*p* < 0.05). Notably, the Standard AU group showed significantly higher expression levels of *MUC2* and *OCLN* compared to the AU group (*p* < 0.05). However, no significant difference in *ZO-1* expression was observed between the Standard AU and AU groups (*p* > 0.05). Interestingly, *CLDN1* expression in the AU group tended to be higher than in the Standard AU group. These results suggest that both the Standard AU and AU extracts can repair the intestinal barrier in intestinal epithelial cells by regulating the expression of tight junction proteins and mucins.

### 3.9. Effect of AU on DSS-Induced Inflammatory Factors in IPEC-J2 Cells

As shown in Figure 7E–G, DSS significantly increased the expression of pro-inflammatory factors *IL-18* and *TNF-α* (*p* < 0.05) and significantly decreased the expression of the anti-inflammatory factor *IL-10* (*p* < 0.05) compared to the CON group. In contrast, both the Standard AU and AU groups significantly reduced the expression of *IL-18* and *TNF-α* (*p* < 0.05) and significantly increased the expression of *IL-10* (*p* < 0.05) compared to the DSS group. Notably, the expression of *IL-10* in the Standard AU group was significantly higher than in the AU group (*p* < 0.05). However, no significant difference was observed in the expression of *IL-18* between the Standard AU and AU groups (*p* > 0.05). Additionally, *TNF-α* levels in the AU group displayed an upward trend relative to those in the Standard AU group. These results suggest that both Standard AU and AU extracts can reduce intracellular inflammation by upregulating anti-inflammatory factors and downregulating pro-inflammatory factors, thereby restoring cellular homeostasis.

### 3.10. Effect of AU on Genes Related to MyD88/NF-κB Signaling Pathway in DSS-Induced IPEC-J2 Cells

In Figure 7H–M, the expression levels of *IL-1β*, *IL-1R*, *MyD88*, *TAK1*, *IKKα*, and *RelA* were significantly higher in the DSS group compared to the CON group (*p* < 0.05). However, the expression levels of these inflammatory factors were significantly reduced in both the Standard AU and AU groups (*p* < 0.05). Notably, the Standard AU group demonstrated a restoration of *IL-1R*, *TAK1*, and *RelA* expression levels to near-normal levels (*p* > 0.05). In this experiment, AU inhibited the release of *IL-1β* and reduced inflammation. Therefore, in DSS-induced IPEC-J2 cells, the anti-inflammatory effect of AU may in-volve inhibition of the MyD88/NF-κB signaling pathway. This hypothesis is based on indirect evidence from downstream inflammatory factors (such as reduced expression of *IL-1β* and *TNF-α*). Future studies may further validate this through Western blot analysis.

## 4. Discussion

To investigate potential treatments for colitis, many studies utilize chemical in-ducers such as TNBS, DSS, or OXA to induce colitis in mice, which closely mimic the human inflammatory process [18]. Among these, DSS is one of the most widely used agents due to its adjustable concentrations, flexible administration cycles, ability to consistently induce model states, short experimental duration, and high success rate. Depending on the experimental goals, researchers can choose protocols to establish either acute or chronic ulcerative colitis (UC) models. Acute UC models are commonly established by offering unrestricted access to drinking water supplemented with 2%–5% DSS (molecular weight 36–50 kDa) over 5–7 days. For example, Nunes et al. [19] induced acute UC in C57BL/6 mice with a 2% DSS solution for 7 days, while Jin et al. [20] used a 2.5% DSS solution for the same duration in the same strain. In the present study, mice were administered 3% DSS in drinking water ad libitum for 3 days, followed by a repeat of this cycle for five iterations. Current first-line treatments for inflammatory bowel disease (IBD) include aminosalicylates, corticosteroids, immunosuppressants, and biologics [21]. However, these therapies have safety concerns and limited efficacy, which has led to a growing interest in natural extracts as alternative treatments. For instance, Wang et al. [22] explored the therapeutic effects of grape seed proanthocyanidin extract (GSPE) in treating recurrent UC in rats, while Xin et al. [23] examined the protective effects of camellia seed shell polysaccharides on DSS-induced colitis in mice. Pomari et al. [24] studied the effect of AL extract in treating UC. Given these findings, this study focused on aucubin, an active iridoid glycoside derived from *Eucommia ulmoides*, and evaluated its therapeutic effects on DSS-induced intestinal inflammation.

In the present investigation, relative to the model group, the AU group reduced the DAI score in mice and alleviated the trend of weight loss. Huang et al. [25] documented comparable results in their research examining AL’s protective role against DSS-triggered UC in murine models, indicating that AL significantly influences both the DAI score and weight changes. Additionally, mice in the DSS group exhibited increased organ indices, shorter colon lengths, structural damage, loss of goblet cells, and severe ulcers in the mucosal layer. Studies have shown that when the body is stimulated, organs may undergo pathological changes such as congestion, hyperplasia, and hypertrophy, leading to an increase in the organ index [26]. Using DSS for modeling induces intestinal inflammation and spasm, resulting in shortened colon length and structural damage [27]. However, after AU treatment, all of these symptoms were alleviated, which is consistent with other research findings. Crocin and Centella Asiatica glycosides can improve DSS-induced structural damage to the mouse colon by protecting the intestinal barrier [28,29].

An imbalance in oxidative stress is a hallmark of IBD. Key antioxidants, such as GSH-Px and CAT, help neutralize hydrogen peroxide, thus counteracting oxidative stress [30]. Superoxide dismutase (SOD), a vital metalloenzyme, plays a critical role in maintaining redox balance. Lipid peroxidation, which increases membrane permeability, can lead to significant tissue damage, and malondialdehyde (MDA) levels serve as a direct indicator of lipid peroxidation severity [31]. Pomari et al. [24] examined the impact of AL extract on UC management. This investigation revealed that AL can increase the activity of antioxidant enzymes, such as GSH and SOD, re-duce LPO, and prevent the formation of reactive ROS. Xue et al. [32] et al. reported reduced SOD activity in IBD models and highlighted that active compounds in honeysuckle enhanced its activity. In the present study, aucubin demonstrated protective effects against IBD in mice by upregulating antioxidant enzyme activities (e.g., SOD, GSH-Px, CAT), reducing MDA levels, and alleviating oxidative damage.

Research has confirmed that pro-inflammatory cytokines such as *IL-1β*, *TNF-α*, *IL-18*, along withsignaling molecules like MyD88 and NF-κB, play central roles in regulating inflammatory responses and have been established as core biomarkers in DSS-induced inflammatory bowel disease (IBD) models. The development of IBD is closely associated with the overexpression of these inflammatory mediators.The gut mucosal barrier—formed by intestinal epithelial cells along with their inter-connecting tight junctions—serves an essential function in preserving intestinal bal-ance and shielding against microbial threats. By interacting with the gut microbiota, immune cells, and exosomes, the mucosal barrier forms a robust line of defense between the intestine and the external environment, thereby preserving intestinal integrity [33]. The barrier function of the intestine is essential for overall health, and tight junction proteins such as *OCLN*, *CLDN1*, *CLDN2*, and *ZO-2* serve as key markers of mucosal permeability and structural integrity [34]. Mucin 1 (*MUC1*), secreted by intestinal epithelial cells, is a critical component of the chemical barrier, contributing to lubrication of the intestinal lining and prevention of bacterial adhesion. During the progression of IBD, the expression of inflammatory factors increases significantly, while the intestinal barrier becomes severely compromised. Eucommia glycoside significantly downregulates the expression of pro-inflammatory cytokines and upregulates the expression of intestinal barrier proteins such as *OCLN*, *CLDN2*, and *MUC1*, thereby contributing to the maintenance of intestinal health. Our observations accord with the results reported by Qiu et al. [35], indicating that luteolin attenuates proinflammatory cytokine levels in IBD murine models and bolsters anti-inflammatory molecule production.

In a healthy body, the microbiota coexists with the host and its immune system in a balanced state, enabling both the coexistence of commensal bacteria and an appropriate response to intestinal pathogens. However, this interaction is disrupted in patients with IBD [36]. The microbiota consists of four major phyla: *Proteobacteria*, *Actinobacteria*, *Firmicutes*, and *Bacteroidetes*, each comprising thousands of species. While bacterial diversity varies due to factors such as diet, medication, time, and others, multiple studies have shown that overall microbial diversity decreases during the development of IBD [37,38]. Gut dysbiosis, defined by shifts in microbial composition, diversity, spatial distribution, and population density, stands as a defining feature of inflammatory bowel disease (IBD) [39]. In this study, IBD induced dysbiosis in the intestinal microbiota, leading to an overgrowth of harmful bacteria such as *Helicobacter*, *Escherichia*, and *Shigella*. At the genus level, eucommoside modulated the intestinal microbiota by upregulating the abundance of beneficial bacteria, including *Bacteroidetes*, *Firmicutes*, and *Verrucomicrobium*, while downregulating the abundance of *Proteobacteria* and *Deferribacterota*. These results align with findings documented in previous investigations. For instance, Liu et al. [40] observed a notable rise in *Helicobacter* pylori levels within the guts of mice exposed to DSS, contributing to mucosal harm. Similarly, Wu et al. [41] showed that lipopolysaccharide (LPS) treatment resulted in a marked reduction in the Vibrio genus in the mouse intestine, which was alleviated by the addition of oyster peptides.

Porcine intestinal epithelial cells (IPEC-J2) are a non-tumorigenic intestinal epithelial cell line commonly used as an in vitro model for studying intestinal cellular immune function, apoptosis, and barrier function [42]. Cytotoxicity, a subfield of toxicology, is used to evaluate food safety and study the activity of components in functional foods [43], often employing methods such as CCK-8 or MTT assays for detection. When intestinal epithelial cells are exposed to external stimuli, it can lead to the dysregulation of cell proliferation and apoptosis mechanisms, compromising cellular integrity, inducing the secretion of inflammatory factors, and ultimately causing inflammatory damage [44]. In this study, DSS-induced inflammation in IPEC-J2 cells was used to reflect the anti-inflammatory effects of AU, as assessed by changes in intestinal barrier function and the expression of inflammatory factor genes.

A well-maintained intestinal structure can resist the invasion of harmful substances. *MUC2*, primarily produced by intestinal goblet cells, serves as the first natural barrier against external harmful substances [45]. *ZO-1* interacts with transmembrane proteins to link tight junctions with the cytoskeleton, forming a stable tight junction structure [46]. *OCLN* deficiency leads to changes in intestinal barrier permeability, whereas *CLDN1* serves as a key component within the tight junction protein family [47]. The intestinal tract functions as the chief venue for nutrient breakdown and uptake, while also acting as a defensive shield, essential for bodily protection. When pathogens or other harmful substances stimulate the body, it triggers self-protective responses, with inflammation being one such response [48]. Thus, this research examined AU’s anti-inflammatory properties through assessment of pertinent gene expressions. The results showed that, compared to the CON group, DSS disrupted the expression of intestinal barrier genes and increased the expression levels of genes related to inflammatory factors. In contrast, AU regulated the expression of intestinal barrier proteins, upregulated the expression of anti-inflammatory genes, downregulated inflammatory factor expression, and reduced DSS-induced inflammatory responses. Similar studies have shown that yellow tea reduces pro-inflammatory cytokine levels through the TLR4-NF-κB-NLRP3 signaling pathway [49]. Kulhari et al. [50] found that biotin may also regulate inflammation by affecting the phosphorylation of NF-κB (p65) protein and reducing inflammatory cytokines, which is consistent with the conclusions of this study. 

In this study, DSS-induced intestinal and cellular inflammation in mice was in-duced, and multiple measured indicators were discussed, demonstrating the efficacy of AU in alleviating inflammation. Limitations of this study include the absence of direct comparison with standard therapies, which may constrain the assessment of AU’s rel-ative efficacy. Future research should include head-to-head comparisons to evaluate its potential for application in health supplements.

## 5. Conclusions

In summary, aucubin can reduce the disease activity index score in IBD mouse models by restoring intestinal barrier function through increased expression of intes-tinal barrier-related genes, alleviating intestinal inflammation by decreasing the ex-pression of inflammatory factors, and modulating the intestinal microbiota by upreg-ulating the abundance of bacterial phyla such as Bacteroidota, Firmicutes, and Verru-comicrobiota while downregulating the abundance of phyla such as Proteobacteria and Deferribacterota. Additionally, AU exhibits similar effects on DSS-induced inflamma-tion in IPEC-J2 cells. This investigation offers foundational evidence for employing aucubin in ulcerative colitis (UC) therapy, highlighting its extensive prospects in func-tional food formulation. However, further development requires pharmacokinetic and toxicity studies to confirm its safety and suitability. Future work should focus on these aspects to bridge basic research with health product applications.

## Figures and Tables

**Table 1:** DAI Scoring Criteria.

Score	Percentage Weight Loss %	Degree of Diarrhea	Degree of Bleeding in the Stool
0	0	Normal	Normal
1	1~5%	Mildly soft stools, loose and shapely	Normal
2	6~10%	Severe soft stools, loose	Small amount of blood in the stool
3	11~20%	Mild diarrhea, loose, moist	Mild blood in stool
4	>21%	Severe diarrhea, liquid adherence to anus	Severe blood in stool

## Data Availability

The data presented in this study are available on request from the corresponding author due to privacy concerns.

## Supplementary Materials

The following supporting information can be downloaded at: https://www.mdpi.com/article/10.3390/foods14213648/s1, Table S1: Primer sequences; Table S2: Primer sequence.
