# Supplementary material for: Protective Effects of Aucubin in DSS-Induced Colitis: Modulation of Inflammatory Pathways, Intestinal Barrier Integrity, and Gut Microbiota
Source: Foods. 2025 Oct 26;14(21):3648. doi: 10.3390/foods14213648 (PMC12610033; doi:10.3390/foods14213648)
Supplement: Supplementary file 1 [file foods-14-03648-s001.zip › foods-3909585-supplementary.pdf]

Table S1. Primer sequences

Gene	Nucleotide sequence of primers (5′-3′)	Product length	NCBI accession number
β-actin	F：CCTAGGCACCAGGGTGTGAT<br>R：AGCACAGGGTGCTCCTCA	200	NM_007393.5
IL-1β	F：AATGCCACCTTTTGACAGTGATG<br>R：AGCTTCTCCACAGCCACAAT	189	NM_008361.4
IL-18	F：ACTTTGGCCGACTTCACTGT<br>R：GGGGTTCACTGGCACTTTGA	126	NM_001357221.1
TNF-α	F：GACGTGGAACTGGCAGAAGAG<br>R：TTGGTGGTTTGTGAGTGTGAG	228	NM_013693.3
NF-κB	F：ATGGCAGACGATGATCCCTAC<br>R：TGTTGACAGTGGTATTTCTGGTG	111	XM_006501107.3
MyD88	F：TCATGTTCTCCATACCCTTGGT<br>R：AAACTGCGAGTGGGGTCAG	175	NM_010851.3
OCLN	F：GGAGGACTGGGTCAGGGAAT<br>R：TCAGCAGCAGCCATGTACTC	198	NM_001360536.1
CLDN1	F：GGGGACAACATCGTGACCG<br>R：AGGAGTCGAAGACTTTGCACT	100	NM_016674.4
CLDN2	F：CCTCGCTGGCTTGTATTATCTCTG<br>R：GAGTAGAAGTCCCGAAGGATG	175	XM_006528487.4
ZO-2	F：ATGGGAGCAGTACACCGTGA<br>R：TGACCACCCTGTCATTTTCTTG	176	NM_001360392.1
MUC1	F：ACCTGGAAGGCCCAATCAAG<br>R：CTCAGCGTAGTTGGCACTCT	111	NM_013605.2

Table S2. Primer sequence

Gene	Nucleotide sequence of primers (5′-3′)	（bp） Product length	NCBI accession number
GAPDH	F：TCGGAGTGAACGGATTTGGC<br>R：TGCCGTGGGTGGAATCATAC	147	NM_001206359.1
ZO-1	F：GAAATACCTGACGGTGCTGC<br>R：GAGGATGGCGTTACCCACAG	147	XM_021098856.1
MUC2	F：AGCTCCAGAGAGAAGGCAGA<br>R：CTCAGGTGCACAGCGAACTC	164	XM_021082584.1
OCLN	F：TAATGGGCGTCAACCCAACA<br>R：TACAATGGCAATGGCCTCCT	164	NM_001163647.2
CLDN1	F：CAACCCGTGCCTTGATGGTA<br>R：GCAACTAAGATAGCCAGACCTGA	172	NM_001244539.1
IL-10	F：AGACGTAATGCCGAAGGCAG<br>R：ACCCCTCTCTTGGAGCTTGC	184	NM_214041.1
TNF-α	F：GGCCCAAGGACTCAGATCAT<br>R：CTGTCCCTCGGCTTTGACAT	82	NM_214022.1
IL-18	F：TGTAGCTGAAAACGATGAAGACC<br>R：AACACGGCTTGATGTCCCTG	123	NM_213997.1
IL-1β	F：AAGATGACACACCCACCCTG<br>R：TGGTTCTGCTCCCAGACTTTC	150	X74568.1
IL-1R	F：CAGGGGTTCCCCTCTCCTAA<br>R：AAGGAATGCATCCACCCAGC	183	XM_021087128.1
MyD88	F：GGCCCAGCATTGAAGAGGA<br>R：TCGAATGGTGATGCCTGACA	125	NM_001099923.1
TAK1	F：AAGGCAAGAGGATGAGTGCT<br>R：TTAGGCTTGGAATAGGCTGTGG	76	NM_001114280.1

IKKα	F：TTTTGGATGGAGTCAGGGGC<br>R：GTGTACTGCTTCGGCCCATA	182	NM_001114279.1
RELA	F：GGCACCGGATTGAGGAGAAA<br>R：GGTCGGTGGGTCCATTGAAA	87	NM_001114281.1
